# Prevalence and Risk Factors of Prehypertension and Hypertension in Southern China

**DOI:** 10.1371/journal.pone.0170238

**Published:** 2017-01-17

**Authors:** Lihua Hu, Xiao Huang, Chunjiao You, Juxiang Li, Kui Hong, Ping Li, Yanqing Wu, Qinhua Wu, Huihui Bao, Xiaoshu Cheng

**Affiliations:** Department of Cardiovascular Medicine, the Second Affiliated Hospital of Nanchang University, Nanchang of Jiangxi, China; Shanghai Institute of Hypertension, CHINA

## Abstract

**Background:**

This study aimed to describe the prevalence and risk factors of prehypertension and hypertension in Jiangxi Province, China. Individuals with prehypertension frequently progress into hypertension and are at high risk of developing cardiovascular disease and stroke.

**Methods:**

A cross-sectional survey of 15,296 participants (15 years or older) was conducted in Jiangxi Province, China, in 2013, using questionnaire forms and physical measurements.

**Results:**

The prevalence of prehypertension and hypertension was 32.3% (39.2% in men and 27.6% in women) and 29.0% (30.1% in men and 28.2% in women), respectively. The awareness, treatment, and control rates among all hypertensive participants were 64.8%, 27.1%, and 12.6%, respectively. The prevalence of prehypertension in males declined with age, but the prevalence of hypertension increased in different genders. The prevalence of prehypertension and hypertension increased with increasing body mass index (BMI). The prevalence of prehypertension decreased, in parallel to an increase in the prevalence of hypertension, with increasing waist circumference (WC). A combination of WC and BMI was superior to individual indices in identifying hypertension. A multivariate logistic regression analysis indicated that increasing age, high BMI, high visceral adipose index, and high heart rate were risk factors for prehypertension and hypertension. The high body fat percentage was significantly associated with prehypertension. Living in an urban area, male sex, abdominal obesity, and menopause were correlated with hypertension.

**Conclusions:**

Prehypertension and hypertension are epidemic in southern China. Further studies are needed to explore an indicator that can represent the visceral fat accurately and has a close relationship with cardiovascular disease.

## 1. Introduction

Hypertension is not only a well-known risk factor for cardiovascular disease but also a public health challenge worldwide [1]. More than 1.5 billion individuals are estimated to currently have hypertension [1–3]. Studies have indicated that blood pressure (BP) values of 120–139/80–89 mm Hg are associated with an increased risk of cardiovascular morbidity and mortality compared with BP levels below 120/80 mm Hg [4–6]. The concept of prehypertension has been defined for a detailed study of the risks of elevated BP. The Seventh Report of the Joint National Committee on Prevention, Detection, Evaluation, and Treatment of High Blood Pressure (JNC-7) proposed high BP category, including 120–139 mm Hg systolic BP (SBP) or 80–89 mm Hg diastolic BP (DBP), designated as prehypertension [7]. According to JNC-7, individuals with prehypertension have a higher risk of developing hypertension compared with those with ideal BP levels; also, they have an increased risk of cardiovascular morbidity and mortality [5–9]. Previous studies have shown that prehypertension is related to a 1.7-fold increase in coronary artery disease and a 3.5-fold increase in myocardial infarction [10]. Moreover, prehypertension is often closely linked to target organ damage, such as early arteriosclerosis, small vascular damage, coronary artery calcification, vascular remodeling, and left ventricular hypertrophy [11–13].

The prevalence of prehypertension and hypertension has significantly increased with rapid economic development, urbanization, acceleration of population aging, and changes in traditional dietary habits and lifestyle in China. Although many studies have focused on prehypertension and hypertension in China [14–18], still marked ethical and geographical differences exist in the prevalence of both hypertension and prehypertension. Moreover, no large-scale surveys have been conducted on the prevalence of prehypertension and correlates of prehypertension and hypertension in southern China, especially Jiangxi Province. Also, previous studies mostly focused on the relationship between smoke, body mass index (BMI), abdominal obesity, and hypertension. Few studies discussed the relationship between visceral adipose index (VAI), body fat percentage (BFP), and BP. Therefore, the present study aimed to estimate the prevalence and correlates of prehypertension and hypertension. Also, the determinants for prehypertension and hypertension were compared. Moreover, scientific references on the primary prevention and intervention strategies for treating prehypertension and hypertension were provided.

## 2. Methods

### 2.1 Ethics statement

Ethical approval was obtained from the ethics review boards of the Second Affiliated Hospital of Nanchang University and the Fuwai Cardiovascular Hospital (Beijing, China). Written informed consent was obtained from each participant and the guardians on behalf of the minors/children aged 15–18 years enrolled in the study. If the guardians were unable to write, then fingerprinting was used. The ethics committee approved the procedure.

### 2.2 Study design

Four cities in urban areas and four counties in rural areas were selected using the probability proportional to size method, in which two districts or two townships were selected. Then, three communities or villages were chosen within each district and township, respectively, using the simple random sampling (SRS) method [19]. Finally, a given number of participants from each of the 14 gender/age strata (male/female and aged 15–24, 25–34, 35–44, 45–54, 55–64, 65–74, and ≥75 years) were chosen using the SRS method according to the national demographic composition; participants were chosen from communities or villages using the lists compiled from the local government registers of households [19]. The design effect was also considered while estimating the sample size. Assuming a design effect of 2.5 and the prevalence of hypertension of 17.7% among population aged 15 years or older, 15,200 participants were needed to ensure that the average lengths of the 95% confidence interval for the prevalence in the entire population and subpopulation defined by age and gender were less than 0.4% and 1.8%, respectively [19]. As a result, a total of 15,364 participants living in Jiangxi Province for 6 months and aged 15 years or older were randomly selected to participate in this survey from November 2013 to August 2014.

### 2.3 Measurement

Participants were required to complete a standardized questionnaire form through face-to-face interviews with trained staff and physical measurements. The questionnaire was developed by the national coordinating center of the Fuwai Hospital (Beijing, China). The anthropometric examinations included body weight, height, waist circumference, BP, resting heart rate, BFP, and visceral fat rate. Specially assigned people managed the whole quality control to ensure the quality and representativeness of the data.

BP was measured using the Omron HBP-1300 Professional Portable Blood Pressure Monitor (Kyoto, Japan) three times on the right arm supported at the heart level after the participants were allowed to rest for 5 min, with a 30-s interval between measurements. SBP or DBP was defined as the average of the three SBP or DBP readings. The subjects were advised to avoid cigarette smoking, and consumption of coffee, tea, and alcohol for at least 30 min before BP measurements. Body weight without heavy clothing, BFP, and VAI were measured using an Omron body fat and weight measurement device (V- BODY HBF-371, Omron, Kyoto, Japan). Height was measured without shoes using a standard right-angle device and a fixed measurement tape (to the nearest 0.5 cm). Waist circumference was measured (to the nearest 0.5 cm) by putting the measuring tape at the midpoint between the lower margin of the last rib and the top of the hip bone (at the level of umbilicus) at the end of expiration.

### 2.4 Definitions

BP classification was based on the JNC7 guidelines [7]. Normotension was defined as subjects with SBP <120 mm Hg and DBP <80 mm Hg without antihypertensive drugs. Prehypertension was defined as not being on antihypertensive drugs and having an SBP of 120–139 mm Hg and/or DBP of 80–89 mm Hg. Hypertension was defined as SBP ≥140 mm Hg and/or DBP ≥90 mm Hg, and also if the individual was on antihypertensive drugs for 2 weeks. BMI was calculated as the weight in kilograms divided by height in meters squared (kg/m^2^). Overweight and obesity were defined as BMI 24–27.9 kg/m^2^ and BMI ≥28 kg/m^2^, respectively [20]. Waist circumference (WC) was divided into abdominal overweight (85–95 cm in males and 80–90 cm in females) and abdominal obesity groups (WC ≥95 cm in males and ≥90 cm in females) [20]. VAI was categorized into three groups as standard (1–9), slightly high (10–14), and high (15–30) [21]. BFP was categorized into four groups as thin (<10% for males and <20% for females), standard (10–19% for males and 20–29% for females), slightly high (20–25% for males and 30–35% for females), and high (≥25% for males and ≥35% for females) [22]. Awareness of hypertension was defined as self-report of any previous diagnosis of hypertension by a health care professional. Treatment of hypertension was defined as self-reported use of antihypertensive medications in the previous 2 weeks among hypertensive participants, as well as among hypertensive participants who were aware of being hypertensive. Among hypertensive participants who were under treatment, control was defined as a systolic BP <140 mm Hg and a diastolic BP <90 mm Hg. Cigarette smokers were defined as having smoked at least one cigarette per day for 6 months or more. Alcohol use was defined as drinking alcohol at least one time per week during the previous year.

### 2.5 Statistical analysis

All data were established using EpiData version 3.02 software. After alignment correction, a statistical analysis was performed using the Statistical Package for Social Science software 17.0 (SPSS, IL, USA) and Microsoft Excel 2007. Continuous variables were presented as mean ± standard deviation or median (IQR) as appropriate and compared using the *t* test or the Mann—Whitney *U* test, which depended on whether the quantitative data were consistent with the normal distribution. Categorical variables were expressed as percentages and analyzed by the chi-square test or Fisher’s exact test as appropriate. Multivariate logistic regression analysis was performed to evaluate significant risk factors for prehypertension (vs normotension), hypertension (vs normotension), and hypertension (vs prehypertension). A *P* value less than 0.05 was considered statistically significant.

## 3. Results

**Fig 1:**
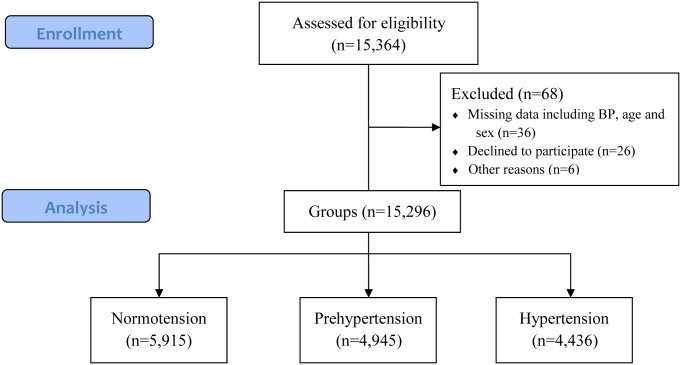
Disposition of patients who were recruited, underwent the groups, and analysed. Sixty-eight participants were excluded because of missing data including sex, age, BP, and so on.

As shown in S1 Table, a total of 15,296 participants from 15,364 eligible participants (6,279 males and 9,017 females; aged 15–97 years) were included in the statistical analysis. Of those, 7805 participants came from urban areas and 7491 from rural areas. Sixty-eight participants were excluded because of missing data including sex, age, BP, and so on. The majority of nonresponders were young people because of their busy work (Fig 1).

### 3.1 Prevalence of prehypertension and hypertension

The baseline characteristics of the study participants stratified by BP category are shown in Table 1. The prevalence of normotension, prehypertension, and hypertension was 38.7% (5915 cases), 32.3% (4945 cases), and 29.0% (4436 cases), respectively. As shown in Table 1, the prevalence of normotension, prehypertension, and hypertension was different among the gender, region, BFP, and VAI categories (*P* < 0.001). Prehypertension group had intermediate levels of age, BMI, WC, BFP, VAI, SBP, DBP, and heart rate (HR), and these values significantly increased in parallel to BP levels. However, alcohol use was significantly lower in patients having hypertension compared with participants having normotension and prehypertension. The prehypertension group had the biggest number of cigarette smokers among the three groups.

**Table 1:** Characteristics of participants stratified by BP.

variables	Normotension (n = 5915)	Prehypertension(n = 4945)	Hypertension (n = 4436)	Statistic	*P* value
Male *N* (percent)	1932 (32.7)	2459 (49.7)	1888 (42.6)	329.99^a^	<0.001
Female *N* (percent)	3983 (67.3)	2486 (50.3)	2548 (57.4)		
Age (years) M (P25~P75)	45 (32~58)	52 (40~65)	66 (57~74)	3075.14^b^	<0.001
Urban *N* (percent)	2806 (47.4)	2364 (47.8)	2635 (59.4)	175.47^a^	<0.001
Rural *N* (percent)	3109 (52.6)	2581 (52.2)	1801 (40.6)		
Current smokers *N* (percent)	806 (13.6)	1123 (22.7)	835 (18.8)	150.77^a^	<0.001
Current drinkers *N* (percent)	1271 (21.5)	1341 (27.1)	1035(23.3)	47.86^a^	<0.001
BMI (kg/m^2^) M (P25~P75)	21.5 (19.7~23.6)	22.8 (20.6~25.2)	23.7(21.3~26.2)	923.67^b^	<0.001
WC (cm) M (P25~P75)	75.0 (70.0~80.0)	79.4 (73.0~86.0)	82.0(75.4~89.5)	1285.14^b^	<0.001
BFP M (P25~P75)	25.4 (21.0~30.4)	26.5 (21.8~32.0)	29.6 (24.1~35.0)	689.31^b^	<0.001
<10 for M,<20 for F	435(61.1)	176 (24.7)	101 (14.2)	1523.842^a^	<0.001
10–19 for M, 20–29 for F	2819(53.0)	1605 (30.2)	892 (16.8)		
20–24 for M, 30–34 for F	1652 (35.7)	1590 (34.4)	1380 (29.9)		
≥25for M,≥35 for F	897 (20.4)	1527 (34.6)	1983 (45.0)		
VAI M (P25~P75)	5.0 (3.0~8.0)	7.0 (5.0~10.0)	8.0 (6.0~12.0)	1449.05^b^	<0.001
1–9	4909 (44.2)	3501 (31.5)	2705 (24.3)	872.225^a^	
10–14	566 (22.0)	944 (36.7)	1063 (41.3)		
15–30	119 (12.5)	326 (34.2)	508 (53.3)		
SBP (mmHg) M (P25~P75)	110.3(104.5~115.0)	126.3(122.3~131.3)	144.7(134.3~156.3)	11183.62^b^	<0.001
DBP (mmHg) M (P25~P75)	67.3 (62.5~72.0)	76.0 (71.3~81.0)	81.3 (73.0~90.0)	5233.83^b^	<0.001
HR (bpm) (P25~P75)	76 (70~83)	78 (71~85)	78 (71~85)	36.51^b^	<0.001

*Note*: Continuous variables were presented as median (IQR) as appropriate and compared using the Mann—Whitney *U* test. Categorical variables were expressed as percentages and analyzed by the *χ*^2^ test.

Variables are shown as M (P25–P75) or percentage.

^a^ χ^2^ test;

^b^ Mann—Whitney *U* test.

BFP, Body fat percentage; BMI, body mass index; DBP, diastolic blood pressure; HR, heart rate; SBP, systolic blood pressure; WC, Waist circumference. M, Male; F, female.

### 3.2 Prevalence of prehypertension and hypertension stratified by sex and age

The prevalence of prehypertension was 39.2% in males and 27.6% in females. The prevalence of hypertension was 30.1% in males and 28.2% in females. Overall, the prevalence of prehypertension remained closer across younger age groups (<45 years), peaked at the age of 45–54 years, then decreased. The prevalence of hypertension increased with age in both genders (*P* < 0.001). However, the prevalence of prehypertension in males declined (*P* < 0.01), while the prevalence in females remained constant at an age less than 45 years and decreased in older age groups. Hence, the prevalence of prehypertension and hypertension was significantly higher in males than in females. Table 2 presents the prevalence of hypertension in women aged 65 years and older, which was higher than that of men. No difference in the prevalence of hypertension was observed at the age of 65–97 years.

**Table 2:** Prevalence of prehypertension and hypertension stratified by sex and age.

Age (year)	Total (n = 15296)				Female (n = 9017)				Male (n = 6279)				P*value	*P*^&^ value	*P*^Δ^ value
	N	NMT	PHT	HT	N	NMT	PHT	HT	N	NMT	PHT	HT			
Overall^a^	15296	38.7	32.3	29.0	9017	44.2	27.6	28.2	6279	30.7	39.2	30.1	<0.001	0.049	0.469
15~24	1337	65.5	32.6	1.9	766	79.1	20.0	0.9	571	47.3	49.6	3.1	<0.001	<0.001	0.416
25~34	1334	61.9	32.8	5.3	758	77.3	19.1	3.6	576	41.5	50.9	7.6	<0.001	<0.001	0.904
35~44	2087	56.7	33.7	9.6	1254	67.7	25.1	7.2	833	40.2	46.7	13.1	<0.001	<0.001	0.904
45~54	2934	41.7	37.6	20.7	1861	46.2	34.3	19.5	1073	34.0	43.3	22.7	<0.001	<0.001	0.417
55~64	3068	29.7	32.5	37.8	1858	31.3	31.2	37.5	1210	27.4	34.4	38.2	0.012	<0.001	0.345
65~74	2685	22.0	29.3	48.7	1480	23.2	26.8	50.0	1205	20.5	32.3	47.2	0.005	0.093	0.007
75~97	1851	16.4	26.0	57.6	1040	15.2	24.8	60.0	811	17.9	27.5	54.6	0.683	0.514	0.075
*P* value			<0.001	<0.001			<0.001	<0.001			0.009	<0.001			

*Note*: Values are percentages.

HT, Hypertension; NMT, normotension; PHT, prehypertension.

^a^Age-standardized prevalence rate.

* Comparison between prehypertension and normotension in different genders;

^&^ comparison between hypertension and normotension;

^Δ^ comparison between prehypertension and hypertension.

A *P* value of less than 0.017 was considered statistically significant.

### 3.3 Prevalence of prehypertension and hypertension stratified by BMI and WC

A total of 332 participants with missing information on weight, height, and/or waist circumference were not included in the analysis. The prevalence of hypertension increased among those who were overweight and obese in both genders and was even higher for those with abdominal obesity compared with those without abdominal obesity in the same BMI category (Table 3). The highest combination of WC and BMI categories (BMI ≥28 kg/m^2^ and WC ≥95 cm for men and ≥90 cm for women) was associated with a greater risk of having hypertension. The combination of WC and BMI was superior to the individual indices in identifying hypertension. The prevalence of prehypertension decreased in both males and females in the same BMI category with an increase in WC. However, the prevalence of prehypertension increased in both men and women in the same WC category with an increase in BMI. The prevalence of prehypertension and hypertension was significantly different within different BMI and WC categories (*P* < 0.05).

**Table 3:** Prevalence of prehypertension and hypertension stratified by BMI and WC.

WC (cm)	BMI<24 kg/m^2^				24≤ BMI<28 kg/m^2^				BMI≥28 kg/m^2^				P*value	*P*^&^ value
	N	NMT	PTH	HT	N	NMT	PTH	HT	N	NMT	PTH	HT		
Male														
Overall^a^	4022	36.8	38.2	25.0	1589	20.1	41.7	38.2	516	12.8	41.8	45.4	<0.001	<0.001
<85	3578	38.4	38.2	23.4	497	22.9	46.7	30.4	55	20.0	52.7	27.3	<0.001	<0.001
85~	418	23.4	37.6	39.0	913	19.4	40.7	39.9	152	13.8	42.8	43.4	0.042	0.064
95~	26	30.8	34.6	34.6	179	15.6	33.0	51.4	309	11.0	39.5	48.5	0.033	0.023
P value			0.002	<0.001			0.974	<0.001			0.723	0.018		
Female														
Overall^a^	5894	51.5	25.8	22.7	2273	31.3	31.9	36.8	670	21.6	30.6	47.8	<0.001	<0.001
<80	4731	55.8	25.0	19.2	535	38.3	31.4	30.3	63	36.5	38.1	25.4	<0.001	<0.001
80~	1063	34.4	29.9	35.7	1300	31.3	33.5	35.2	178	29.2	33.2	37.6	0.094	0.474
90~	100	25.0	23.0	52.0	438	22.6	27.4	50.0	429	16.3	28.4	55.3	0.070	0.040
*P* value			<0.001	<0.001			0.038	<0.001			0.111	<0.001		

*Note*: Values are percentages.

HT, Hypertension; NMT, normotension; PHT, prehypertension.

^a^ Age-standardized prevalence rate.

* Comparison between prehypertension and normotension in different BMI categories;

^&^comparison between hypertension and normotension in different BMI categories.

### 3.4 Prevalence of prehypertension and hypertension in urban and rural areas

The prevalence of prehypertension in urban and rural areas was 30.3% and 34.5%, respectively, and no significant urban—rural difference was observed in males or females (Table 4). The prevalence of hypertension was 33.7% in urban and 24.0% in rural areas, with a significant difference in both genders (*P* < 0.01). The prevalence of prehypertension and hypertension increased with the increase in BMI and WC in both urban and rural areas. Moreover, the prevalence of hypertension had a statistical difference (*P* < 0.01). No significant urban—rural difference in the prevalence of prehypertension was observed in different ages. However, the prevalence of hypertension in urban areas was significantly higher than that in rural areas with the increase in age.

**Table 4:** Prevalence of prehypertension and hypertension in urban and rural areas.

Variables	Urban (n = 7805)				Rural (n = 7491)				P*value	*P*^&^ value
	N	NMT	PHT	HT	N	NMT	PHT	HT		
Overall	7805	36.0	30.3	33.7	7491	41.5	34.5	24.0	0.703	<0.001
Gender										
Male	3278	29.7	36.2	34.1	3001	31.9	42.4	25.7	0.151	<0.001
Female	4527	40.5	26.0	33.5	4490	47.9	29.1	23.0	0.276	<0.001
BMI										
Normal	4852	43.9	28.2	27.9	5163	47.2	33.2	19.6	0.038	<0.001
Overweight	2172	24.2	33.9	41.9	1712	30.0	38.5	31.5	0.295	<0.001
Obesity	693	15.2	35.4	49.4	512	22.2	35.5	42.3	0.023	0.001
Abdominal obesity										
Yes	871	15.4	31.4	53.2	621	21.4	29.3	49.3	0.010	0.004
No	6803	38.3	30.2	31.5	6765	43.0	35.1	21.9	0.443	<0.001
Age(years)										
15~24	655	66.9	31.0	2.1	682	64.2	34.2	1.6	0.240	0.554
25~34	696	63.3	31.2	5.5	638	60.2	34.6	5.2	0.185	0.991
35~44	907	55.3	33.1	11.6	1180	57.8	34.2	8.0	0.927	0.006
45~54	1234	40.3	34.9	24.8	1700	42.8	39.6	17.6	0.441	<0.001
55~64	1577	27.9	31.3	40.8	1491	31.8	33.7	34.5	0.508	0.001
65~74	1618	19.8	28.5	51.7	1067	25.3	30.5	44.2	0.102	<0.001
75~97	1118	15.1	23.1	61.8	733	18.3	30.4	51.3	0.558	0.004

*Note*: BMI, body mass index; HT, hypertension; NMT, normotension; PHT, prehypertension.

* Comparison between prehypertension and normotension;

^&^comparison between hypertension and normotension.

A total of 192 participants with missing information on weight and/or height and 236 participants with missing information on waist circumference were not included in the analysis.

### 3.5 Prevalence of prehypertension and hypertension stratified by VAI and BFP

**Fig 2:**
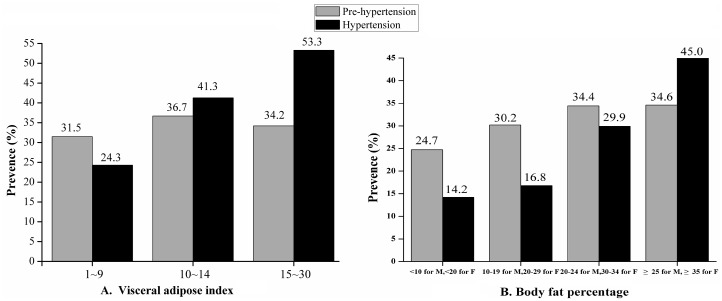
Prevalence of prehypertension and hypertension stratified by VAI and BFP. (A) Prevalence of prehypertension and hypertension stratified by visceral adipose index; (B) Prevalence of prehypertension and hypertension stratified by body fat percentage.

Fig 2 shows the prevalence of prehypertension and hypertension with increased VAI and BFP, respectively. The prevalence of hypertension and prehypertension increased with an increase in VAI (*χ*^2^ = 355.29, *P* < 0.001; *χ*^2^ = 121.23, *P* < 0.001) (Fig 2A). When VAI was ≥10, the prevalence of hypertension was higher than that of prehypertension. Also, the prevalence of hypertension and prehypertension increased with an increase in BFP (*χ*^2^ = 1416.40, *P* < 0.001; *χ*^2^ = 528.38, *P* < 0.001)(Fig 2B). Moreover, the gap between the prevalence of hypertension and prehypertension gradually shortened. When BFP was ≥25% for males and ≥35% for females, the prevalence of hypertension was higher than that of prehypertension.

### 3.6 Awareness, treatment, and control of hypertension

Of the 4436 hypertensive individuals, 2874 (1206 males and 1668 females) were aware of their condition, 1201 (523 males and 678 females) accepted antihypertensive drugs, and 558 (233 males and 325 females) controlled their BP in the normal range (Table 5). On the whole, the awareness, treatment, and control rates among all hypertensive participants were 64.8%, 27.1%, and 12.6%, respectively. The awareness, treatment, or control rate was not significantly different between males and females (*P* > 0.05); however, these parameters were higher in urban areas than in rural areas, with statistical significance (*P* < 0.01). Also, the awareness, treatment, and control rates of hypertension increased with an increase in age till the age of 75 years.

**Table 5:** Awareness, treatment, and control rates of hypertension.

	n	awareness	treatment	control
Location				
Urban, n (%)	2635	1769 (67.1)	889 (33.7)	390 (14.8)
Rural, n (%)	1801	1105 (61.4)	312 (17.3)	168 (9.3)
Gender				
Male, n (%)	1888	1206 (63.9)	523 (27.7)	233 (12.3)
Female, n (%)	2548	1668 (65.5)	678 (26.6)	325 (12.8)
Age (years)				
15~24, n (%)	25	2 (8.0)	1 (4.0)	0.0
25~34, n (%)	71	26 (36.6)	6 (8.5)	4 (5.6)
35~44, n (%)	199	86 (43.2)	28 (14.8)	20 (10.1)
45~54, n (%)	606	350 (57.8)	145 (23.9)	72 (11.9)
55~64, n (%)	1159	755 (65.1)	355 (30.6)	170 (14.7)
65~74, n (%)	1309	928 (70.9)	405 (30.9)	180 (13.8)
75~97, n (%)	1067	727 (68.1)	261 (24.5)	112 (10.5)
Total	4436	2874 (64.8)	1201 (27.1)	558 (12.6)

### 3.7 Risk factors associated with prehypertension and hypertension

A multivariate logistic regression analysis was performed using SPSS to assess significant determinants of prehypertension (vs normotension), hypertension (vs normotension), and hypertension (vs prehypertension). The results are shown in Table 6. People aged 45–54, 55–64, 65–74, and ≥75 years had a greater correlation of developing prehypertension compared with participants aged 15–24 years [odds ratio (OR) = 2.290, 2.875, 3.526, and 5.241, respectively]. The probability of having prehypertension and hypertension increased with an increase in BMI and VAI. Moreover, patients with a high HR were more likely to develop prehypertension and hypertension (*P* < 0.001). Interestingly, the multivariate logistic regression analysis showed a significant increase in ORs with an increase in BFP in the prehypertension group (*P* < 0.01), not in the hypertension group. In contrast, gender, location, smoking, drinking, menopause, abdominal obesity, and low BFP were not significantly associated with prehypertension. Also, a higher correlation of hypertension with increasing age was observed. Compared with females, males were positively associated with hypertension (OR = 2.408). People living in urban areas were also associated with hypertension (OR = 1.317). Smoking and drinking were not significantly associated with hypertension. Unlike prehypertension, menopause, and abdominal obesity were associated with a greater likelihood of having hypertension (OR = 1.334, 1.464). However, taking prehypertension as the reference, increasing age, living in urban areas, male sex, menopause, and abdominal obesity were still associated with hypertension, but no significant differences were found for other risk factors (all *P* > 0.05).

**Table 6:** Factors associated with Pre-hypertension and Hypertension.

Characteristics	Pre-hypertension/Normotension		Hypertension/Normotension		Hypertension/Pre-hypertension	
	OR (95% CI)	*p* value	OR (95% CI)	*p* value	OR (95% CI)	*p* value
Gender(male/Female)	1.454 (0.843, 2.508)	0.178	2.408 (1.370, 4.233)	0.002	1.656 (1.002, 2.737)	0.049
Location(Urban/ Rural)	0.952 (0.851, 1.065)	0.393	1.317 (1.161, 1.494)	0.001	1.383 (1.222, 1.566)	<0.001
Smoking (Yes/No)	1.362 (0.948, 1.957)	0.095	1.250 (0.813, 1.921)	0.310	0.910 (0.619, 1.336)	0.629
Drinking (Yes/No)	1.024 (0.874, 1.200)	0.769	0.858 (0.710, 1.036)	0.112	0.838 (0.695, 1.009)	0.062
Age (years)						
15~24	1.000		1.000		1.000	
25~34	0.939 (0.716, 1.232)	0.650	3.921 (1.479, 10.397)	0.006	4.175 (1.544, 11.293)	0.005
35~44	1.232 (0.965,1.572)	0.094	8.685 (3.477, 21.692)	<0.001	7.050 (2.781, 17.872)	<0.001
45~54	2.290 (1.803, 2.908)	<0.001	29.347 (11.941, 72.123)	<0.001	12.817 (5.152, 31.883)	<0.001
55~64	2.875 (2.162, 3.824)	<0.001	65.241 (26.216, 162.358)	<0.001	22.692 (9.017, 57.106)	<0.001
65~74	3.526 (2.614, 4.757)	<0.001	125.781 (50.432, 313.706)	<0.001	35.674 (14.159, 89.883)	<0.001
75~97	5.241 (3.765, 7.295)	<0.001	251.488(100.013, 632.377)	<0.001	47.985 (18.981, 121.311)	<0.001
Menopause (Yes/No)	1.077 (0.909, 1.277)	0.391	1.334 (1.102, 1.616)	0.003	1.239 (1.022, 1.500)	0.029
BMI						
Normal	1.000		1.000		1.000	
Overweight	1.591 (1.373, 1.845)	<0.001	1.944 (1.650, 2.290)	<0.001	1.221 (1.042, 1.432)	0.014
Obesity	1.750 (1.283, 2.385)	<0.001	2.159 (1.650, 2.290)	<0.001	1.234 (0.924, 1.648)	0.154
Abdominal obesity(Yes/No)	0.933 (0.732, 1.189)	0.574	1.464 (1.152, 1.860)	0.002	1.570 (1.267, 1.945)	<0.001
BFP						
<10 for M,<20 for F	1.000		1.000		1.000	
10–19 for M, 20–29 for F	1.195 (0.916, 1.560)	0.190	0.654 (0.472, 0.907)	0.011	0.547 (0.385, 0.777)	0.001
20–24 for M, 30–34 for F	1.476 (1.113, 1.956)	0.007	1.008 (0.723, 1.405)	0.964	0.683 (0.479, 0.974)	0.035
≥25for M,≥35 for F	1.582 (1.161, 2.155)	0.004	1.082 (0.760, 1.539)	0.663	0.684 (0.473, 0.989)	0.44
VAI						
1–9	1.000		1.000		1.000	
10–14	1.305 (1.038, 1.642)	0.023	1.290 (1.017, 1.637)	0.036	0.988 (0.801, 1.220)	0.914
15–30	2.882 (1.845, 4.500)	<0.001	3.455 (2.129, 5.607)	<0.001	1.199 (0.823, 1.747)	0.344
HR	1.011 (1.005, 1.016)	<0.001	1.014 (1.009, 1.020)	<0.001	1.004 (0.998, 1.009)	0.163

## 4. Discussion

Many epidemiological studies have demonstrated that prehypertension and hypertension are the biggest contributors to the global burden of disease worldwide, and the prevalence of prehypertension and hypertension in different countries and districts differs significantly. The present findings showed that the prevalence of prehypertension was 32.3% among individuals 15 years or older in Jiangxi Province, which was consistent with the rate in the adult population of Zhejiang Province (32.1%) [22] and lower than the rate in northeastern China (36.0%) [14], Taiwanese adults (34.0%) [23], Brazilian adults (36.1%) [24], and southern Iran adults [25]. Also, the overall prevalence of hypertension in this study was 29.0%, which was comparable to that in China (29.6%) [26] and developed countries such as the United States (29.3%) [27], higher than that in Zhejiang Province (24.59%) [22] and inner Mongolia (28.61%) [28], but significantly lower than that reported in previous studies [14,18,24].

The present study also revealed that the prevalence of prehypertension decreased in males with increasing age. However, it peaked at the age of 45–54 years in females, and then decreased with increasing age. The prevalence of hypertension increased with age, especially in individuals aged ≥45 years, which was consistent with the findings of other studies [14,24,26,29]. Overall, the prevalence of prehypertension and hypertension was significantly higher in males than in females. However, the prevalence in females aged ≥65 years was higher than that in males in the same age group because of the rapid increase in prevalence in females compared with males, which was similar to previous reports [30]. Also, the multivariate logistic regression analysis showed a significant increase in the ORs with an increase in age in the prehypertension and hypertension groups. This difference might be related to hormonal changes at different ages in both genders. Previous studies have reported a higher prevalence of hypertension in postmenopausal than in premenopausal women [31–33]. Therefore, frequent monitoring is needed for early detection of hypertension during the menopausal transition. Although estrogen deficiency during menopause may induce endothelial and/or vascular dysfunction through reduced compliance of the large arteries [33], the mechanisms by which BP increases after menopause have not been well characterized and need further exploration.

The improvement in living standards has led to an increase in obesity, especially during adolescence. The prevalence of overweight adults has been reported as high as 17.7% with adult obesity at 5.6% in China [34]. Overweight/Obesity can cause significant insulin resistance, accompanied by a corresponding increase in the prevalence of hypertension, and weight control can significantly lower BP [35]. Some studies showed that overweight, obesity, or central obesity was significantly associated with prehypertension and hypertension [26–28]. The present study found that the prevalence of prehypertension and hypertension increased among those who were overweight and obese in both genders. Similarly, multivariate logistic regression analysis showed that different from hypertension, the prevalence of prehypertension decreased in both genders with the increase in WC. This was because most of the individuals in abdominal obesity groups progressed to actual hypertension, further confirming that abdominal obesity was more related to BMI in predicting the risk factors of hypertension [36,37]. This study also suggested that the combination of WC and BMI was superior to individual indices as a measure for evaluating hypertension. These results indicated once again that modifying lifestyle, such as weight loss, may be an effective way for lowering long-term BP [35].

VAI, located in the abdomen, is a major contributor to abdominal obesity, contrary to subcutaneous fat abundant in the buttocks and lower limbs. Recently, VAI, not total or subcutaneous adiposity, has been proven to be a good predictor of prehypertension and hypertension [38,39]. However, additional studies are needed to elucidate the mechanisms underlying this association. The present study indicated that VAI was more sensitive than abdominal obesity. BFP can also reflect the body fat. Previous studies reported that high BFP was associated with hypertension, and SBP and DBP gradually increased with BFP [22,39]. However, this study indicated that BFP was associated with hypertension and prehypertension. However, the multivariate logistic regression analysis showed that BFP might have a significant correlation with prehypertension, not hypertension. Therefore, the relationship between BFP and hypertension is worth further discussion.

The proportion of rural and urban populations in this study was close to 1:1. The prevalence of prehypertension had no significant urban—rural difference with respect to gender and age. Table 6 shows that the prevalence of hypertension in urban areas was higher than that in rural areas. Living in an urban area had an increased correlation with hypertension (OR = 1.317), which was contrary to the findings of other studies [14,22]. Moreover, the prevalence of hypertension in urban and rural areas increased rapidly with an increase in age, BMI, or WC, and the former was significantly greater than the latter. Therefore, the prevalence of hypertension in urban areas was obviously higher than that in rural areas in relation to age and obesity.

The awareness, treatment, and control rates among all hypertensive participants were 64.8%, 27.1%, and 12.6%, respectively. All parameters were higher than those in China [26]. The improvement was likely to be an effective measure of the prevention and control of hypertension. Besides, people themselves paid more attention to their health. Moreover, the awareness, treatment, and control of hypertension were still poor compared with those in developed countries [40,41]. The present study showed that the awareness, treatment, and control of hypertension in urban areas were better compared with those in rural areas, probably because people living in urban areas had a higher level of education, higher income, and better medical conditions. The result was consistent with other researches [22,26]. Therefore, the government should pay more attention to the rural areas. In another study [22], the awareness, treatment, and control of hypertension in females were higher than those in males; however, no significant differences in both genders were found in the present study. This might be due to the same economic status of both genders. The treatment and control of hypertension in individuals aged 25–34 and 35–44 years were low, which might be because of their busy schedule and bad habits including smoking, drinking, and staying up late. Hence, more effective primary prevention measures should be also taken for the young and middle-aged populations.

Surprisingly, the present survey indicated that smoking and drinking were not associated with prehypertension and hypertension. This result was not comparable to other studies [14,22,26]. The difference might be due to the following reasons. First, the standard of smoking and drinking was not the same. Second, the proportion of smoking and drinking was less. Third, the present study had a higher proportion of females. According to most of the reports, smoking and excessive drinking were the risk factors for prehypertension and hypertension. At the same time, the recommendation of JNC-7 suggested that lifestyle modifications might be necessary for all individuals to prevent prehypertension and hypertension, including reducing alcohol consumption and giving up smoking.

Moreover, high HR was found to be significantly associated with prehypertension and hypertension. Studies showed that hypertensive subjects had higher HRs than normotensive subjects, and an elevated HR was associated with a rise in peripheral BP and increased risk for hypertension [42,43]. However, the guidelines do not consider HR while choosing antihypertensive medications, despite the link between HR and development of hypertension. This is because lowering of HR may reduce peripheral BP, but not reliably reduce central BP.

The present study had several limitations. First, it was a cross-sectional survey and failed to establish a cause-and-effect relationship between risk factors and the development of prehypertension and hypertension. Second, the participants might have the recall bias, resulting in the difference in information provided. Third, due to economical and human resource constraints, data including salt intake, physical activity, and levels of homocysteine, blood lipid, and blood glucose were lacking.

In conclusion, the present study revealed a high prevalence of prehypertension and hypertension in an adult population of Jiangxi Province, but the awareness, treatment, and control of hypertension remained relatively low. It also showed a correlation between BMI and VAI with prehypertension and hypertension, BFP with prehypertension, and abdominal obesity with hypertension. Moreover, further studies are needed to explore an indicator that can represent the visceral fat accurately and has a close relationship with cardiovascular disease.

## Supporting Information

S1 Table Minimal data set. (XLSX)
